# Heme oxygenase induction attenuates TNF-α-induced hypertension in pregnant rodents

**DOI:** 10.3389/fphar.2015.00165

**Published:** 2015-08-17

**Authors:** Eric M. George, Jacob M. Stout, David E. Stec, Joey P. Granger

**Affiliations:** ^1^Department of Physiology and Biophysics, University of Mississippi Medical Center, Jackson, MS, USA; ^2^Department of Biochemistry, University of Mississippi Medical Center, Jackson, MS, USA

**Keywords:** pre-eclampsia, VEGF, TNF-α, sFlt-1, heme oxygenase

## Abstract

Pre-eclampsia is a hypertensive disorder of pregnancy initiated by placental insufficiency and chronic ischemia. In response, several pathways activated in the placenta are responsible for the maternal syndrome, including increased production of the anti-angiogenic protein, sFlt-1, and inflammatory cytokines, especially tumor necrosis factor-alpha (TNF-α). Previous studies have demonstrated that heme oxygenase (HO) induction can block TNF-α pathways *in vitro* and attenuate placental ischemia-induced sFlt-1 *in vivo*. Here, we investigated whether HO-1 induction could attenuate TNF-α-induced hypertension in pregnant rats. In response to TNF-α infusion (100 ng/day i.p.), maternal mean arterial pressure (MAP) increased vs. control animals (104 ± 3 vs. 119 ± 3 mmHg). HO-1 induction had no effect in control animals, but significantly decreased MAP in TNF-α-infused animals (108 ± 2 mmHg). Placental vascular endothelial growth factor (VEGF) was decreased in response to TNF-α infusion (92 ± 4 vs. 76 ± 2 pg/mg). Placental sFlt-1 was increased by TNF-α infusion (758 ± 45 vs. 936 ± 46 pg/mg, *p* < 0.05), which trended to normalization by HO-1 induction (779 ± 98 pg/mg). In contrast, HO-1 induction had no significant effect on placental VEGF in TNF-α-infused animals. Taken together, these data suggest that one of the key mechanisms by which HO exerts cytoprotective actions in the placenta during inflammation due to chronic ischemia is through suppression of sFlt-1. Further work elucidating the bioactive metabolites of HO-1 in innate inflammatory responses to placental ischemia is warranted.

## Introduction

Perhaps the most persistent and pervasive of obstetrical disorders is pre-eclampsia, which occurs in approximately 5% of all births in the United States, is a contributor to up to 15% of all preterm births, and is a leading cause of maternal/fetal morbidity (Meis et al., 1998; Sibai et al., 2005; American College of Obstetricians and Gynecologists and Task Force on Hypertension in Pregnancy, 2013). The classical definition of pre-eclampsia is new-onset hypertension during gestation with proteinuria; however, newer guidelines from the American Congress of Obstetricians and Gynecologists suggest criteria of hypertension coupled with either proteinuria or a broader array of symptoms (thrombocytopenia, renal insufficiency, impaired liver function, pulmonary edema, or cerebral and visual symptoms) could increase the diagnosis of this disorder in the population (American College of Obstetricians and Gynecologists and Task Force on Hypertension in Pregnancy, 2013). Despite intensive research, the underlying causes of pre-eclampsia remain obscure. However, it is believed in most cases, especially the most severe, the underlying pathology is due to placental underperfusion. It is further believed that this is due to the maternal spiral arteries, which feed the placenta to fully remodel and vasodilate, and provide proper blood flow to the developing uteroplacental unit. In response, the placenta becomes hypoxic and ischemic, and begins to release soluble factors that are released into the maternal bloodstream and cause the maternal symptoms of the disorder (Lim et al., 1997; George and Granger, 2012).

The identities of these soluble factors have been a major area of investigation, and several have been repeatedly and strongly implicated in both patients and animal models of placental ischemia, including the anti-angiogenic protein, sFlt-1, and circulating autoantibodies to the angiotensin type 1 receptor (Maynard et al., 2003; Xia et al., 2003). One of the earliest and most consistent findings, however, is the activation of the innate immune response, in particular inflammatory cytokines, such as tumor necrosis factor-alpha (TNF-α) and IL-6 (Vince et al., 1995). This increase in TNF-α and IL-6 has also been observed in animal models of experimental placental ischemia (Gadonski et al., 2006; LaMarca et al., 2008). Infusion of these factors individually during pregnancy in rodents have been shown to induce hypertension, though not to the degree seen in a placental ischemic model, suggesting that they are only partially causative of the phenotype (Alexander et al., 2002; Gadonski et al., 2006). There has been some interest in targeting the TNF-α system as a potential therapeutic approach for the management of pre-eclampsia. There are unanswered questions as to the safety of currently-used recombinant protein and antibody-based TNF-α antagonists despite several ongoing trials assessing their safety during pregnancy (George, 2014).

Several lines of evidence suggest that the detrimental cardiovascular effects of TNF-α elevation could be attenuated by induction of heme oxygenase-1 (HO-1). The predominant function of HO is in the conversion of free heme from heme-containing proteins to biliverdin, which is quickly converted by biliverdin reductase to bilirubin, which can then be conjugated for excretion in the bile. In the process, the biologically-active byproducts, carbon monoxide (CO) and bilirubin, are produced. These in turn are believed to have important roles in blood pressure regulation through anti-inflammatory, antioxidant, and vasorelaxation mechanisms (Cao et al., 2009). HO-1 induction has been shown to be protective and anti-hypertensive in several experimental models of hypertension, including the reduced uterine perfusion pressure (RUPP) model, known to be associated with elevated TNF-α levels (Sabaawy et al., 2001; Yang et al., 2004; Botros et al., 2005; George et al., 2011b). Early reports suggested that placental HO-1 induction could also protect the placenta from TNF-α-induced cytotoxicity, perhaps preserving placental function (Ahmed et al., 2000). Here, we test the hypothesis that HO-1 induction can attenuate the hypertension associated with elevated circulating TNF-α, and then determined the effects of both on pro- and anti-angiogenic proteins in pregnant rats.

## Materials and Methods

### Animals

Timed pregnant Sprague Dawley rats (Harlan, Indianapolis, IN, USA) were received on gestational day 11, held at a constant 23°C, and put on a 12:12-h light-dark cycle with food and water *ad libitum*. All protocols were approved by the University of Mississippi Medical Center Institutional Animal Care and Use Committee and followed the National Institutes of Health Guidelines for the Care and Use of Laboratory Animals.

### TNF-α Administration and HO-1 Induction

On gestational day 14, animals in the experimental groups were implanted i.p. with mini-osmotic pumps containing recombinantly-expressed TNF-α (R&D Systems, Minneapolis, MN, USA) or vehicle alone. TNF-α was received lyophilized and reconstituted in sterile PBS. Mini-pumps (Model 2001, Alzet, Cupertino, CA, USA) delivered TNF-α at a constant dose of 100 ng/day. Briefly, rats were anesthetized and maintained with 3% isoflurane. A ventral midline incision ∼3 cm in length was made, and pumps were placed i.p. The incision was closed with non-absorbable continuous sutures. Animals were euthanized on gestational day 19 and tissues harvested post-mortem. For HO-1 induction, on gestational day 14, cobalt protoporphyrin IX chloride (CoPP, Frontier Scientific, Logan, UT, USA) was injected i.p. at a dose of 5 mg/kg, which we have previously shown to cause persistent elevation in HO-1 production in the placenta (George et al., 2011a,b). Each experimental group consists of 7–8 animals.

### Measurement of Arterial Pressure

On gestational day 18, animals were anesthetized as above, and implanted with indwelling carotid catheters. The catheter was externalized at the nape of the neck s.c.. The following day, animals were placed in restraint cages and acclimatized. Mean pressure was determined over the course of 30 min via direct pressure transducers (ADInstruments, Bella Vista, NSW, Australia).

### Tissue Harvest

Rats were anesthetized as above. The uterus was externalized thorough a ventral midline incision, and blood was collected by cannulation of the abdominal aorta for isolation of plasma and serum. Records were made of the viable and resorbed pups present in each animal, and individual pups and placentas were weighed and recorded. Representative placental samples from each horn, thoracic aortae, and livers were flash frozen in liquid nitrogen and stored at –80°C for later analysis.

### Measurement of sFlt-1, VEGF, and TNF-α

Placental protein was prepared from a representative placenta from individual rats. Briefly, the frozen tissue was mechanical ground by mortar and pestle in liquid nitrogen. Tissue fragments were resuspended in radioimmunoassay buffer with protease inhibitor cocktail, phenylmethanesulfonyl fluoride (PMSF), and sodium orthovanadate (Santa Cruz Biotechnology Inc., Dallas, TX, USA). Homogenization was performed via automated tissue processor (MP Biomedicals, LLC, Santa Ana, CA, USA) and the solution was cleared by centrifugation at 12000 × g for 20 min. Resulting protein concentrations were measured by the bicinchoninic acid method (Pierce Biotechnology, Rockford, IL, USA). Vascular endothelial growth factor (VEGF) and sFlt-1 were both measured using sandwich ELISA (R&D Systems, Inc., Minneapolis, MN, USA) in duplicate according to manufacturer’s instructions. Measurements of free plasma VEGF were performed with the same kit. Plasma TNF-α levels were measured using a commercial ELISA per manufacturer’s instructions (R&D Systems, Inc.).

### Statistical Analyses

All date are displayed as means ± SEM. Comparisons between groups were performed by one-way ANOVA with Tukey’s multiple comparisons test at a significance threshold value of *p* < 0.05. All statistical comparisons and graphs were generated with Prism 6 (GraphPad, La Jolla, CA, USA).

### HO Activity Assays

Determination of total HO activity (*n* = 5 in each group) in liver and placenta was performed as described previously (Vera et al., 2007). Briefly, for HO activity, tissue lysates were prepared and measured as above. Reactions were performed in 1.2 mls consisting of: 2 mM glucose-6-phosphate, 0.2 units glucose-6-phosphatedehydrogenase, 0.8 mM NADP, 20 μM hemin, and 0.5 mg of protein from lysates. Incubations were allowed to proceed for 1 h at 37°C. Resulting bilirubin was chloroform extracted and concentration was determined by change in optical density at 464–530 nm, utilizing an extinction coefficient of 40 mM/cm. Activity was expressed as pmol bilirubin formed/hr/mg of protein. Five samples were analyzed in each group.

## Results

### Chronic TNF-α Infusion Leads to Increased Circulating Levels of TNF-α

**FIGURE 1:**
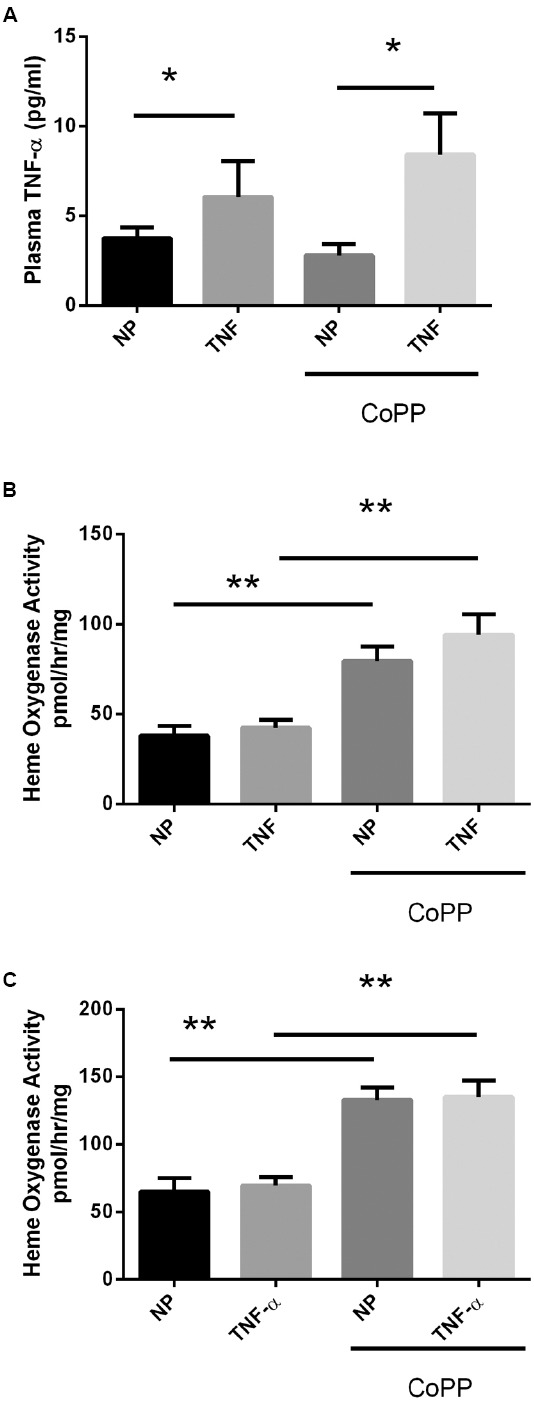
**Circulating plasma TNF-α levels were determined by ELISA (A).** In response to continuous i.p. TNF-α infusion (TNF), plasma levels were elevated in both normal pregnant (NP) and CoPP-treated animals. There was no statistical difference in circulating TNF-α levels between control or CoPP-treated animals. Statistical significance at *p* < 0.05 is indicated by connecting lines. **(B)** Liver and placenta **(C)** heme oxygenase activity was determined in response to TNF-α infusion and CoPP administration. TNF-α infusion alone had no effect on heme oxygenase activity, however, CoPP administration significantly increased HO activity in both control and TNF-α infused animals. Level of significance is indicated by asterisks (*p < 0.05, **p < 0.005).

To determine whether the administered dose of TNF-α was sufficient to exert a biological effect, plasma levels of the protein were determined via sandwich ELISA. Control animals exhibited low circulating levels of TNF-α (Figure 1A; 3.8 ± 0.2 pg/mL). Animals receiving TNF-α demonstrated a significant ∼50% increase in circulating TNF-α levels (6.1 ± 0.9 pg/mL, *p* < 0.05). Administration of CoPP had no significant effect on the circulating levels of TNF-α in either control (2.8 ± 0.4 pg/mL) or TNF-α-infused rats (8.4 ± 2.3 pg/mL). Taken together, these data demonstrate that TNF-α infusion increases circulating levels of TNF-α, while CoPP administration has no direct effect. The efficacy of CoPP for induction of HO activity was ascertained by examining HO activity in the livers and placentas of all groups. As seen in Figures 1B,C, TNF alone had no effect on HO activity, but increased HO activity in both control and TNF-infused animals.

### HO-1 Induction Attenuates TNF-α-induced Increases in Blood Pressure Without Adverse Effects on Gross Fetal and Placental Development

**FIGURE 2:**
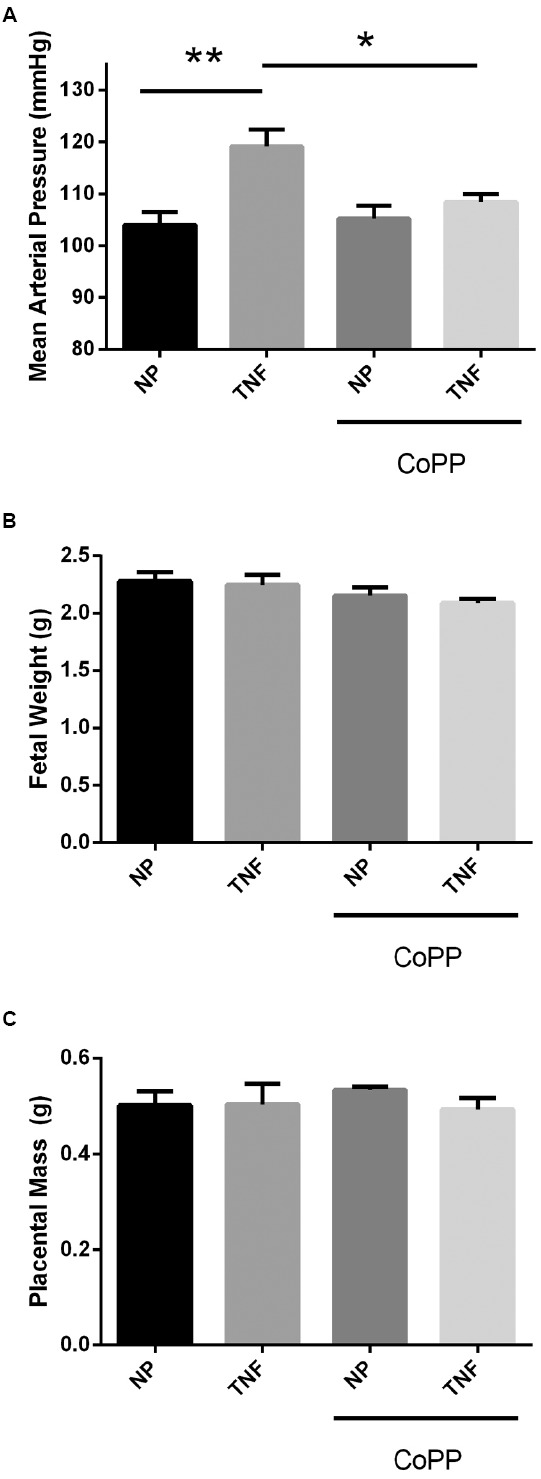
**(A)** In response to TNF-α infusion, mean arterial pressure was significantly elevated compared to normal pregnant controls. Administration of CoPP had no significant effect on mean pressure in normal pregnant animals, but significantly reduced the mean pressure in animals infused with TNF-α. There was no significant effect of either TNF-α infusion or CoPP treatment on either fetal weight **(B)** or placental mass **(C)**. Statistical significance at *p* < 0.05 is indicated by connecting lines. Level of significance is indicated by asterisks (*p < 0.05, **p < 0.005).

After 5 days of continuous infusion of TNF-α (Figure 2A), pregnant dams exhibited a significant 15-mmHg increase (104 ± 3 vs. 119 ± 3 mmHg, *p* < 0.005) in mean arterial pressure (MAP). While administration of the HO-1 inducer CoPP had no effect on control animals (104 ± 3 vs. 105 ± 3 mmHg), it significantly attenuated the rise in blood pressure caused by infusion of TNF-α (119 ± 3 vs. 108 ± 2 mmHg, *p* < 0.05). These data suggest that induction of HO-1 has no effect on blood pressure under normal conditions in pregnant rats, but blocks the hypertensive effects of TNF-α during pregnancy. In line with other published data in pregnant rodents, there was no effect of HO-1 induction on either fetal or placental mass (Figures 2B,C).

### HO-1 Induction Has Differential Effects on Placental VEGF and sFlt-1 Levels

**FIGURE 3:**
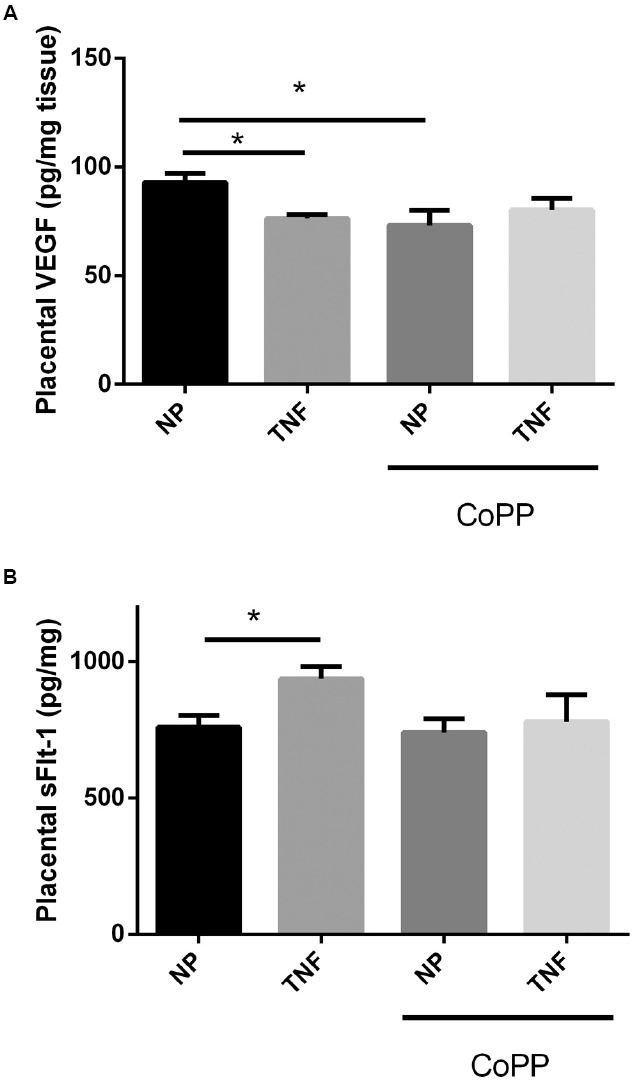
**Infusion of TNF-α led to a small, but significant, decrease in placental VEGF (A), as did administration of CoPP.** There was no statistically significant difference between TNF-α-infused animals with or without CoPP administration. TNF-α infusion was associated with an increase in placental sFlt-1 **(B)**. CoPP had no effect on placental sFlt-1 in normal pregnant animals, but trended to normalization of sFlt-1 levels in TNF-α-infused animals, though this failed to meet significance (*p* = 0.09). Statistical significance at *p* < 0.05 is indicated by connecting lines. Level of significance is indicated by asterisks (*p < 0.05).

To determine the effects of TNF-α infusion and HO-1 induction on the pro-angiogenic protein VEGF and its antagonist sFlt-1, placental levels of both were determined by sandwich ELISA. Interestingly TNF-α infusion resulted in a decrease in placental VEGF (92 ± 4 vs. 76 ± 2 pg/mg, *p* < 0.05; Figure 3A), and an increase in placental sFlt-1 (758 ± 45 vs. 936 ± 46 pg/mg, *p* < 0.05; Figure 3B). In control animals, while HO-1 induction had no significant effect on placental sFlt-1 (Figure 3B); there was significant, decrease in placental VEGF (93 ± 4 vs. 73 ± 7 pg/mg, *p* < 0.05; Figure 3A). Induction of HO-1 in TNF-α-infused animals likewise had no effect on placental VEGF. There was, however, a strong trend toward normalization of placental sFlt-1 levels, but was not statistically significant (936 ± 46 vs. 779 ± 98 pg/mg, *p* = 0.09).

## Discussion

Despite intensive research into the central causes and molecular mechanisms responsible for the maternal pathology of pre-eclampsia, little in the way of new therapeutic approaches has been translated into the clinical setting. It is now widely believed that defects in placental development, specifically failure to adequately remodel the maternal vasculature feeding the placenta, are a key central mechanism in the development of the disorder (Lim et al., 1997; George and Granger, 2012). In response, several key pathways activated in response to placental ischemia have been identified in the maternal symptomatic response; key among them being angiogenic imbalance, innate immune activation, production of oxidative stress, and adaptive immune responses. HO and its metabolic byproducts have been shown to affect several of these pathways independently. HO, CO, and bilirubin have all been shown to down-regulate sFlt-1 production both *in vivo* and *in vitro* (Cudmore et al., 2007; George et al., 2011b, 2012). Bilirubin is a potent antioxidant, which demonstrates a beneficial effect in hypoxia-induced superoxide production, and has been shown to attenuate angiotensin II-induced hypertension experimentally (Vera and Stec, 2010; George et al., 2012; Stec et al., 2013). Recently, we have utilized two models of experimental hypertension in rodents to determine the beneficial effects of HO-1 induction. In the first, we use the RUPP rodent model, which induces placental ischemia in late gestation and shares many features of severe pre-eclampsia. Induction of HO-1 in these animals not only attenuated the hypertension, but also normalized angiogenic balance and placental superoxide production (George et al., 2011b). In the second model, we used the sFlt-1 infusion model, which clamps circulating sFlt-1 at an elevated level. Here too, hypertension was attenuated, which suggests HO-1 activity independent of sFlt-1 suppression (George et al., 2011a). One pathway known to be activated in response to placental ischemia (in which HO-1 induction has not been adequately described *in vivo*) is the induction of inflammatory cytokines—TNF-α in particular.

Elevation of TNF-α has been one of the most consistent findings in both pre-eclamptic patients and experimental forms of placental ischemia-induced pre-eclampsia models (Kupferminc et al., 1994; Vince et al., 1995; LaMarca et al., 2008). Previously, it has been reported that infusion of TNF-α to pregnant rodents to roughly equivalent levels seen in pre-eclamptic patients results in hypertension, though the degree of hypertension displayed is not equivalent to that seen in placental ischemia models (Alexander et al., 2002; LaMarca et al., 2005; Gadonski et al., 2006). Importantly, though antagonism of TNF-α by administration of a soluble receptor tended to decrease the hypertension associated with placental ischemia, this was not significant, suggesting that its hypertensive effects are only a part of a much broader response (LaMarca et al., 2008).

Several previous reports have supported a potential cytoprotective effect of HO-1 in response to TNF-α exposure. Using placental tissue explants, Ahmed et al. (2000) first demonstrated that HO-1 induction, via hemin exposure, was capable of protecting cells from TNF-α-induced cytotoxicity. More recently, Pullikotil et al. (2012) suggested that induction of HO-1 was the mechanism by which epigallocatechin suppressed the inflammatory activity of TNF-α in vascular endothelial cells. Finally, Ndisang recently demonstrated that induction of HO-1 in the spontaneously-hypertensive rat resulted in significantly lower levels of a number of inflammatory cytokines, including TNF-α and IL-6 (Ndisang, 2014). While TNF-α has been shown to stimulate placental sFlt-1 expression, there are no reports on whether induction of HO-1 attenuates TNF-α-induced sFlt-1 production.

Here, we have attempted to examine directly the effects of HO-1 induction in a TNF-α infusion model of hypertension in rodents during late gestation. As a consequence of TNF-α infusion, circulating levels of the cytokine were elevated approximately 50% compared to controls. These levels were not affected by the induction of HO-1 by CoPP in either control or TNF-α groups. Consistent with previously published reports in pregnant rodent models, infusion of TNF-α resulted in a significant increase in blood pressure of 15 mmHg. Induction of HO by CoPP was measured by enzymatic activity in both liver and placenta, and was found to be elevated in both tissues in both control and TNF-α infused animals at similar levels. HO induction, which had no effect on blood pressure in control animals, returned blood pressure to levels undistinguishable from controls in TNF-α animals. Promisingly, this had no significant detrimental effects on either placental or fetal weight, suggesting that HO-1 induction had no negative effect on placental efficiency. Interestingly, TNF-α infusion was associated with increased placental levels of sFlt-1 and decreased levels of VEGF. In both cases, induction of HO-1 caused trends to normalization of both proteins, though neither reached statistical significance. Together, these data suggest that, among other potential pathways, one of the mechanisms by which HO-1 induction could be attenuating the effects of TNF-α in response to placental ischemia is through modulation of sFlt-1 and/or VEGF.

Still unknown is the underlying molecular mechanism by which HO-1 is eliciting this effect. As it has been previously reported that CO suppresses sFlt-1, it is possible that this is the bioactive component at work here (Cudmore et al., 2007; George et al., 2012). Whether the signaling of the CO, the antioxidant effects of bilirubin, or other undetermined effects are responsible are still open fields for investigation. Further work examining the contributions of the individual metabolites to this therapeutic effect should prove enlightening.

### Conflict of Interest Statement

The authors declare that the research was conducted in the absence of any commercial or financial relationships that could be construed as a potential conflict of interest.
